# A novel trophectoderm biopsy technique for all blastocyst stages

**DOI:** 10.1002/rmb2.12418

**Published:** 2021-10-04

**Authors:** Yamato Mizobe, Yukari Kuwatsuru, Yuko Kuroki, Yumiko Fukumoto, Mari Tokudome, Harue Moewaki, Mia Watanabe, Tokiko Iwakawa, Kazuhiro Takeuchi

**Affiliations:** ^1^ Takeuchi Ladies Clinic/Center for Reproductive Medicine Aira‐shi Japan

**Keywords:** blastocyst, extrusion method, next‐generation sequencing, preimplantation genetic testing, trophectoderm biopsy

## Abstract

**Purpose:**

This study was conducted to assess the effectiveness of a new trophectoderm (TE) biopsy method that does not require prior opening of the zona pellucida at the blastocyst stage.

**Methods:**

TE biopsy was conducted using a modified extrusion method for embryos during the cleavage stage. In this method, culture medium was injected into the perivitelline space to help extrude TE cells from the zona pellucida before TE biopsy.

**Results:**

Our extrusion method preserves the embryo culture environment until immediately before biopsy because it does not require opening of the zona pellucida prior to TE biopsy. Furthermore, this method does not require a waiting time for blastocyst hatching after laser irradiation, thereby minimizing damage to the embryos and maintaining the time schedule of culture operations.

**Conclusions:**

TE biopsy using this novel extrusion method may be useful in various applications, including the collection of TE cells for next‐generation sequencing analysis.

## 1 INTRODUCTION

To increase the success rate of *in vitro* fertilization, the rate of implantation of embryos harboring numerical chromosomal abnormalities must be limited while ensuring implantation of euploid embryos. Currently, the most effective method for selecting euploid embryos involves preimplantation genetic testing for aneuploidy (PGT‐A). Considering that 60% of spontaneous abortions and stillbirths result from aneuploidy, testing for these chromosomal abnormalities can greatly reduce the rate of pregnancy loss.^1^ Specifically, the reported correlation among the incidence of miscarriage, aneuploid embryos, and maternal age at delivery highlights the effectiveness of PGT‐A screening.^2^ However, PGT‐A requires the collection of biopsied cells from preimplantation embryos. Although conventional biopsies are commonly performed on embryos during the cleavage stage, this strategy only permits the examination of one or two cells, leading to an increased risk of misdiagnosis because of the high incidence of embryo mosaicism at the cleavage stage.^3^ Another disadvantage of this method is the maldevelopment of embryos, with an associated low pregnancy rate as a result of a low ratio of the number of biopsy specimens to total cells.^4^, ^5^ Because of this, our hospital has adopted a modified extrusion method (m‐Ext method) involving a modified version of the original extrusion method (o‐Ext method)^6^ for the biopsy of embryo blastomeres during the cleavage stage of development; this method is combined with fluorescence *in‐situ* hybridization to diagnose aneuploid embryos. Moreover, recent advances in genetic analysis techniques have led to the combination of array‐comparative genomic hybridization, next‐generation sequencing (NGS), and PGT of trophectoderm (TE) cells in blastocysts, with most recent approaches shifting toward NGS. Additionally, the use of TE biopsies at the blastocyst stage allows for multiple collections of cell specimens.

**FIGURE 1:**
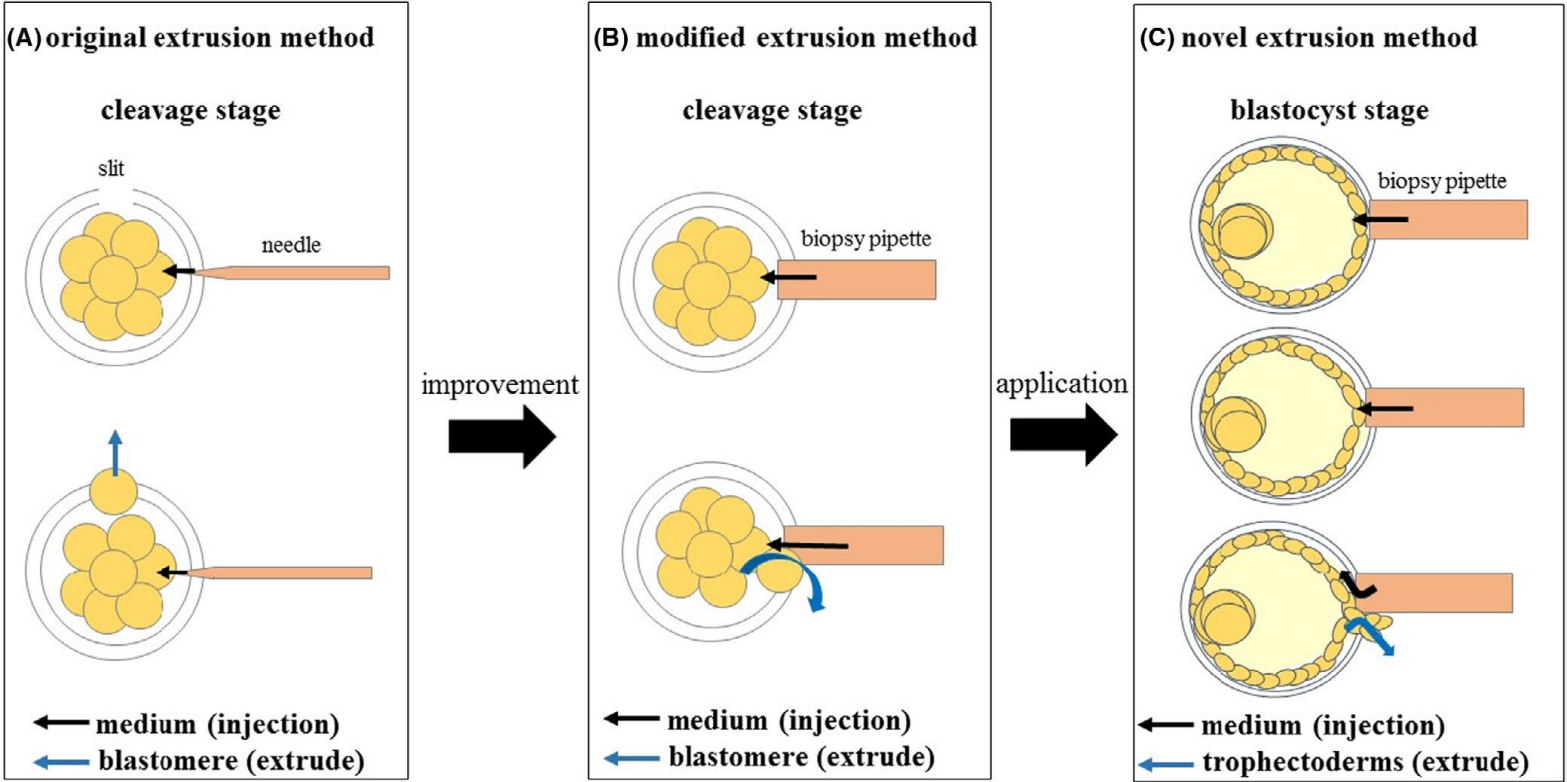
Scheme of each method. (A) Original extrusion method: The insertion point of the media and ejection point of the blastomeres are different. (B) Modified extrusion method: The insertion point of the media and ejection point of the blastomeres are the same. (C) Novel extrusion method: The insertion point of the media and ejection point of the trophectoderms are the same

The m‐Ext method involves creating an opening in the zona pellucida by laser irradiation. Culture medium is then injected to the perivitelline space to extrude blastomeres, which eject from the opening point used for culture injection. Thus, the m‐Ext method differs from the o‐Ext method, i.e., the former has the same injection and ejection points of medium and blastomeres, respectively, while the latter has different injection and ejection points (Figure 1). In this study, we have used a novel extrusion method (n‐Ext) for embryos that had developed to blastocysts without prior zona pellucida opening, which is used for biopsy at the cleavage stage. We compared this method to the conventional TE biopsy method for embryos developing into hatching blastocysts by opening the zona pellucida in advance.

## 2 MATERIALS AND METHOD

### 2.1 Ovarian stimulation

Oocyte retrieval was performed at the Takeuchi Ladies Clinic between January 2016 and January 2020. During the oocyte retrieval cycle, ovarian stimulation was performed using standard gonadotropin‐releasing hormone agonist follicle‐stimulating hormone protocols or an antagonist follicle‐stimulating hormone protocol. Oocytes were retrieved under the guidance of transvaginal ultrasound 36 h post‐injection of human chorionic gonadotropin (Fuji Pharmaceutical Company, Ltd.).

### 2.2 Embryo culture

Cumulus oocyte complexes obtained using the oocyte retrieval procedure described above were preincubated for 3 h (in a gas phase of 5% O_2_, 6% CO_2_, and 89% N_2_ at 37°C), followed by denuding of cumulus/oocyte complex cells. Oocytes in metaphase II were used for intracytoplasmic sperm injection and then cultured in an EmbryoScope + time‐lapse incubator (Vitrolife). Fertilization was confirmed 16–18 h post‐intracytoplasmic sperm injection, after which blastocysts developed over a maximum 6‐day incubation period in Global medium (LG; Life Global) while covered with mineral oil (SAGE, Origio). Biopsies were performed on blastocysts that patients had elected to destroy.

### 2.3 Conventional TE biopsy method

Biopsy was performed on TE cells of blastocysts, taking care to avoid the inner cell mass (ICM). After installing a holding pipette (Medi‐Con International) and biopsy pipette with an internal tip diameter of 25 μm in the injection holder (Sunlight Medical), blastocysts were transferred to the biopsied tissue (Falcon 351007, Corning, Inc.). The biopsy pipette was washed with polyvinyl pyrrolidone (Orgio) to coat the inside of the pipette and prevent cell attachment. Two types of conventional biopsy methods were performed. The first involved creating an opening in the zona pellucida of the embryos before they had reached the blastocyst stage on day 3 or 4. The second method involved artificially shrinking the blastocyst^7^ to widen the perivitelline space. Next, laser irradiation was applied partially to the zona pellucida. In both methods, embryos that reached the blastocyst stage were fixed at the 9 o'clock position using the holding pipette, and TE cells were collected, avoiding the ICM. Unlike biopsies of embryos at the cleavage stage, biopsies at the blastocyst stage, during which cells are adjacent to each other, commonly involve cutting of the TE cells by laser irradiation after suctioning the required TE cells into the biopsy pipette (pulling–stretching method) or using a scraping motion against the holding pipette (flicking method). In this study, we primarily employed the pulling–stretching method for laser irradiation.

### 2.4 Laser irradiation of the zona pellucida

The creation of an opening in the zona pellucida, biopsy, and assisted hatching (AHA) were performed using the LYKOS laser perforation system (Hamilton Thorne, Inc.). Creation of an opening in the zona pellucida was performed using laser irradiation with a heat range (pulse of 300 μs) that minimized damage to the cells.

### 2.5 Clinical application of the n‐Ext method in TE biopsy

**FIGURE 2:**
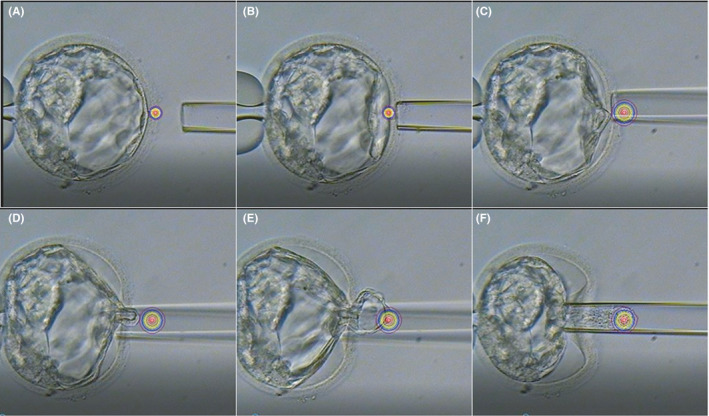
Practical application of the n‐Ext method for trophectoderm (TE) biopsy. (A) Laser irradiation (90 μs) used to create a small opening in the zona pellucida. (B) Culture medium is injected through the opening to enlarge the perivitelline space. (C) Opening of the zona pellucida is increased by laser radiation (90–120 μs) while maintaining the perivitelline space. (D) Medium is injected into the opening to remove TE cells from the inner surface of the opening of the zona pellucida. (E) TE cells are extruded from the opening. (F) After the extruded TE cells are suctioned into the biopsy pipette to obtain required numbers of TE cells, biopsy is performed

Biopsies performed using the n‐Ext method can be conducted without creating an opening in the zona pellucida prior to extrusion and on non‐hatching blastocysts. In embryos suspended with the holding pipette, a small opening was created in the zona pellucida (at a site containing more TE cells) via laser irradiation (90 μs) from the 3 o'clock position. Culture medium was injected into the perivitelline space through the biopsy pipette to remove TE cells from the inner surface of the zona pellucida. The small opening of the zona pellucida was gradually expanded by applying laser irradiation (120 μs) until the diameter was approximately equivalent to that of the biopsy pipette. Culture medium was then injected into the zona pellucida to extrude TE cells from the opening, which were collected as described for the conventional method (Figure 2).

### 2.6 Tubing and NGS analysis of cell specimens obtained in TE biopsy

Tubing and NGS analysis of the cell specimens obtained by TE biopsy were performed as described by Takeuchi et al.^8^

### 2.7 Statistical analysis

We used BellCurve (version 3.20) for Excel (Social Survey Research Information Co., Ltd) for all statistical analyses. Statistical analyses were performed using the Kruskal–Wallis analysis of variance (Table 1). A *p*‐value <0.05 was considered to indicate statistically significant results.

**TABLE 1:** Effect of different biopsy methods on implementation time

Biopsy method	*n*	Age	Time (s)^a^	No. of cells	Survival rate (%)^b^
Conventional	13	42.00 ± 2.68	64.00 ± 17.58^*^	5.69 ± 1.60	13/13 (100.0)
Novel	8	41.17 ± 3.54	129.25 ± 37.30^**^	6.00 ± 1.41	8/8 (100.0)

Note

*^,^** Values with different superscripts are significantly different (*p* < 0.05).

^a^ Time required to complete the biopsy after fixing blastocysts with a holding pipette.

^b^ Survival rate 2–3 h after completion of biopsy.

## 3 RESULTS

**FIGURE 3:**
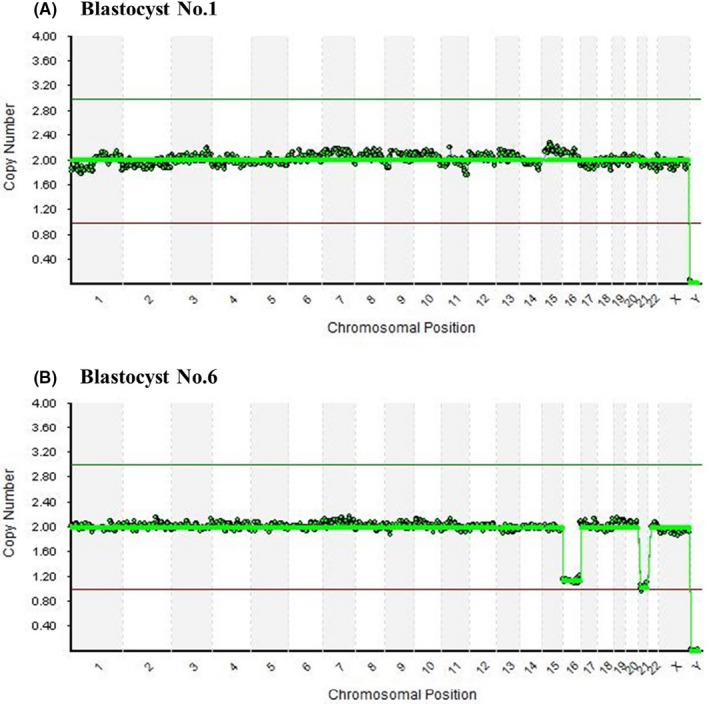
Representative next‐generation sequencing results for biopsy samples obtained using the n‐Ext method. (A) Euploidy (Table 2 Blastocyst No 1), (B) Aneuploidy (Table 2 Blastocyst No 6)

When comparing the required operation time for collecting biopsies between the two methods, the protocol for the n‐Ext method was significantly longer (129.25 ± 37.30 s) compared to the conventional method (64.00 ± 17.58 s) (*p* < 0.05). There was no significant difference in maternal age (41.17 ± 3.54 vs. 42.00 ± 2.68 years, respectively) or the number of collected cells (5.69 ± 1.60 vs. 6.00 ± 1.41, respectively) between the two groups. After biopsy, all blastocysts were viable (Table 1). Furthermore, when performing NGS analysis on samples obtained via the n‐Ext method, all cells were found to be stable (Table 2). Representative charts are presented in Figure 3.

**TABLE 2:** Results of NGS analysis using the n‐Ext method for TE biopsy

Blastocyst no.^a^	Blastocyst grade	No. of cells^b^	Diagnosis	Age
1	4BA	4	Euploidy	39
2	4BC	4	Aneuploidy	46
3	4BB	8	Aneuploidy	42
4	4BB	5	Aneuploidy	42
5	4BA	6	Euploidy	35
6	4BB	5	Aneuploidy	35
7	4BA	6	Aneuploidy	41
8	4BB	6	Aneuploidy	41
9	4BB	8	Aneuploidy	44
10	4CC	3	Aneuploidy	44
11	4BB	3	Euploidy	43
12	4CB	6	Aneuploidy	42
13	4AB	9	Aneuploidy	42
14	4BB	8	Aneuploidy	43
15	4BB	7	Aneuploidy	35

^a^ Mean age of patients was 40.93 ± 3.45 years.

^b^ Mean number of collected cells was 5.87 ± 1.88.

The mean number of cells collected by biopsy using the conventional method was 5.69 ± 1.60, which is similar to the 4–5 cells collected by Kokkali et al.^9^ using this method, whereas that in the biopsy using the n‐Ext method was 6.00 ± 1.41 in the comparative (conventional vs. novel) study and 5.87 ± 1.88 for NGS analysis.

## 4 DISCUSSION

Unlike the conventional method, which can only be applied for the biopsy of hatching blastocysts, the n‐Ext method is advantageous because it can be used for the biopsy of expanded blastocysts before hatching.

There are two types of conventional methods for blastocyst biopsy (i.e., methods for creating an opening in the zona pellucida before TE biopsy); the first involves creating an opening in the zona pellucida of embryos before the blastocyst stage (day 3 or 4), at which point it is difficult to identify the precise position of the ICM, and therefore, this method is associated with an increased risk of ICM hatching, making it challenging to perform this method accurately. The second method involves artificial shrinkage and irradiation of the zona pellucida, thereby necessitating recovery culture. Therefore, this method requires both the creation of an opening in the zona pellucida and regular observation of embryos during daily culture operations. However, regular observation increases the level of environmental exposure experienced by the blastocysts. Thus, observation using a time‐lapse incubator may be necessary.

**FIGURE 4:**
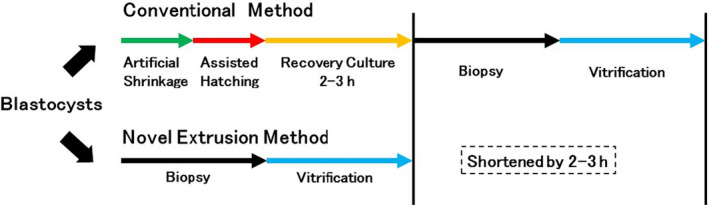
Schematic illustration of the time course of conventional and n‐Ext methods

In contrast, TE biopsy using the n‐Ext method does not require prior creation of an opening in the zona pellucida, eliminating the need for recovery culture. This means that TE biopsy can be performed without waiting for the blastocysts to become available for biopsy (blastocyst hatching). Typical recovery for blastocyst hatching requires 2–3 h, providing a schedule that is more suitable for subsequent procedures (Figure 4). Moreover, TE biopsy using the n‐Ext method may reduce unnecessary observations, thereby maintaining the culture environment and minimally affecting the success of subsequent steps, besides biopsy.

The results of NGS analysis also showed that the n‐Ext method ensures the collection of sufficient cells for further analysis. However, this method also required an extended duration to complete the biopsy procedure (approximately 2 min) compared with the conventional method, as collection of TE biopsies before blastocyst hatching takes a longer time period. In contrast, biopsy collection using the conventional extrusion method is relatively short (approximately 1 min). However, the conventional method also requires 1 min to create an opening in the zona pellucida prior to the hatching process. Hence, the overall *in vitro* exposure times between the two methods were comparable. Another disadvantage of the novel extrusion method is that blastocysts occasionally shrink during the operation. In such cases, the biopsy must be completed before complete shrinkage by inserting the pipette deep into the zona pellucida.

Rubino et al.^10^ reported that AHA was performed in approximately one‐fourth of the zona pellucida after TE biopsy. In fact, they reported the safe completion of AHA after biopsy because of blastocyst shrinkage. Similarly, we generated an opening comprising approximately one‐fifth to one‐fourth of the zona pellucida in blastocysts after biopsy using laser irradiation (120–150 μs). Considering the safety of the embryo in terms of invasiveness, after TE biopsy, the blastocysts are shrunken and AHA can be safely performed on the embryos.

Because of the time lag between biopsy collection and acquiring the test results, the embryos must often be subjected to a freeze–thaw cycle after biopsy. However, if the procedure required for collecting a biopsy from the embryos is highly invasive, blastocysts may not recover after thawing. To assess the viability of blastocysts after being subjected to the n‐Ext biopsy method, we previously performed a biopsy on frozen–thawed blastocysts using this method. The blastocysts were refrozen and thawed using a CryoTip, leading to the recovery of all blastocysts.^8^ In the present study, all blastocysts survived after biopsy (Table 1). Additionally, there was no decrease in the grade of the blastocysts after recovery culture. These results demonstrate that biopsies performed via the novel extrusion method did not affect the recovery of frozen–thawed blastocysts. Furthermore, a good pregnancy rate has been reported with refrozen–thawed embryos using a CryoTip,^11^ suggesting the usefulness of performing biopsy using the n‐Ext method and freeze–thawing with a CryoTip.

In this study, the number of cells varied from three to nine. One of the possible reasons for three‐cell collection was that the TE grade was C. Because embryo survival is lower after biopsy when taking large numbers of cells, only a few cells should be collected. Even when three cells were collected, the results of NGS analysis could be determined. Although a small number of cells may affect NGS analysis, we obtained satisfactory results because we performed NGS in our clinic, which allowed us to control the temperature and eliminate the negative effects of cell transport.

Additionally, performing PGT on blastocysts can interfere with the successful development of embryos (leading to test cancellation) or the absence of euploid embryos.^12^ In fact, blastocysts with good morphology can harbor numerous chromosomal abnormalities,^13^ and transferable embryos may not be obtained. Previous studies showed that PGT using fluorescence *in situ* hybridization does not improve the clinical outcomes of implantation or live births.^14^ Another study reported that cleavage‐stage biopsy significantly reduces the embryonic potential as compared to TE biopsy, which had no negative impact.^15^ Additionally, PGT‐A based on single‐nucleotide polymorphism testing improves clinical outcomes for each transfer site, without affecting oocyte retrieval.^16^ Several studies also demonstrated the effectiveness of using TE biopsy to improve pregnancy rates.^17^, ^18^, ^19^ Preventing aneuploid embryo implantation effectively reduces the risk of unnecessary abortions, leading to fewer treatment cycles and reduced costs, highlighting the advantages of PGT.^20^, ^21^, ^22^

Taken together, our results demonstrate that biopsies performed using the novel extrusion method minimize the impact on daily operations by eliminating the need for recovery culture. In addition, this method enables collection of biopsies during the blastocyst stage before hatching, as it does not required prior laser irradiation. Therefore, biopsies using the novel extrusion method may facilitate the collection of optimal cells required for NGS analysis to ensure accurate and stable results.

## CONFLICT OF INTEREST

The authors declare that they have no conflict of interest.

## ETHICAL APPROVAL

The study was performed with the approval of the institutional review board of the Takeuchi Ladies Clinic. Opt‐out information was posted on a hospital bulletin board.

## HUMAN RIGHTS STATEMENTS AND INFORMED CONSENT

All procedures were performed in accordance with the ethical standard of the responsible committees on human experimentation (institutional and national) and with the Helsinki Declaration of 1964 and its later amendments. Informed consent was obtained from all patients in the study.
